# Carpachromene Ameliorates Insulin Resistance in HepG2 Cells via Modulating IR/IRS1/PI3k/Akt/GSK3/FoxO1 Pathway

**DOI:** 10.3390/molecules26247629

**Published:** 2021-12-16

**Authors:** Rania Alaaeldin, Iman A. M. Abdel-Rahman, Heba Ali Hassan, Nancy Youssef, Ahmed E. Allam, Sayed F. Abdelwahab, Qing-Li Zhao, Moustafa Fathy

**Affiliations:** 1 Department of Biochemistry, Faculty of Pharmacy, Deraya University, Minia 61111, Egypt; rania.alaadin@deraya.edu.eg; 2 Department of Pharmacognosy, Faculty of Pharmacy, South Valley University, Qena 83523, Egypt; emanabdelraheem@svu.edu.eg; 3 Department of Pharmacognosy, Faculty of Pharmacy, Sohag University, Sohag 82524, Egypt; heba.ali@pharm.sohag.edu.eg; 4 Department of Clinical Pathology, Faculty of Medicine, Minia University, Minia 61512, Egypt; n.youssef@worc.ac.uk; 5 Department of Pharmacognosy, Faculty of Pharmacy, Al-Azhar University, Assiut 71524, Egypt; ahmedallam@azhar.edu.eg; 6 Department of Pharmaceutics and Industrial Pharmacy, College of Pharmacy, Taif University, Taif 21944, Saudi Arabia; sayed.awahab@mu.edu.eg; 7 Department of Radiology, Graduate School of Medicine and Pharmaceutical Sciences, University of Toyama, Toyama 930-0194, Japan; 8 Department of Biochemistry, Faculty of Pharmacy, Minia University, Minia 61519, Egypt; 9 Department of Regenerative Medicine, Graduate School of Medicine and Pharmaceutical Sciences, University of Toyama, Toyama 930-0194, Japan

**Keywords:** carpachromene, HepG2 cells, insulin resistance, insulin receptor, PI3K/Akt/GSK3/FoxO1 pathway

## Abstract

Insulin resistance contributes to several disorders including type 2 diabetes and cardiovascular diseases. Carpachromene is a natural active compound that inhibits α-glucosidase enzyme. The aim of the present study is to investigate the potential activity of carpachromene on glucose consumption, metabolism and insulin signalling in a HepG2 cells insulin resistant model. A HepG2 insulin resistant cell model (HepG2/IRM) was established. Cell viability assay of HepG2/IRM cells was performed after carpachromene/metformin treatment. Glucose concentration and glycogen content were determined. Western blot analysis of insulin receptor, IRS1, IRS2, PI3k, Akt, GSK3, FoxO1 proteins after carpachromene treatment was performed. Phosphoenolpyruvate carboxykinase (PEPCK) and hexokinase (HK) enzymes activity was also estimated. Viability of HepG2/IRM cells was over 90% after carpachromene treatment at concentrations 6.3, 10, and 20 µg/mL. Treatment of HepG2/IRM cells with carpachromene decreased glucose concentration in a concentration- and time-dependant manner. In addition, carpachromene increased glycogen content of HepG2/IRM cells. Moreover, carpachromene treatment of HepG2/IRM cells significantly increased the expression of phosphorylated/total ratios of IR, IRS1, PI3K, Akt, GSK3, and FoxO1 proteins. Furthermore, PEPCK enzyme activity was significantly decreased, and HK enzyme activity was significantly increased after carpachromene treatment. The present study examined, for the first time, the potential antidiabetic activity of carpachromene on a biochemical and molecular basis. It increased the expression ratio of insulin receptor and IRS1 which further phosphorylated/activated PI3K/Akt pathway and phosphorylated/inhibited GSK3 and FoxO1 proteins. Our findings revealed that carpachromene showed central molecular regulation of glucose metabolism and insulin signalling via IR/IRS1/ PI3K/Akt/GSK3/FoxO1 pathway.

## 1. Introduction

Insulin resistance is a condition that arises from the defect in insulin-mediated actions on glucose and lipid metabolism, which occurs mainly in liver, muscles, and adipose tissues. This contributes to numerous human diseases including type 2 diabetes mellitus and cardiovascular diseases [1,2]. The causes underlying insulin resistance are a combination of genetic and environmental factors (e.g., diet and exercise). Adipose tissues predominantly contribute to insulin resistance. The limitation of adipose tissue expansion in response to large food intake leads to a high level of circulating lipids, particularly free fatty acids (FFAs) [3]. Insulin exerts its physiological action through binding to and activation of the plasma membrane-bound insulin receptor on target cells. Once activated, it initiates a downstream signaling cascade to phosphorylate the tyrosine residues of the insulin receptor substrate (IRS) family for their activation. IRS-1 (IRS1) and -2 (IRS2) are implicated in most of the metabolic actions mediated via insulin receptors [4]. The activated IRS proteins can further phosphorylate and activate phosphoinositide-3-kinase (PI3K) which in turn results in protein kinase B (Akt) phosphorylation and activation [5,6]. Insulin receptor activation and the consecutive phosphorylation/activation of signaling proteins, primarily IRS1, IRS2, PI3K, and Akt, are largely implicated in insulin action and response at target tissues [1]. Defects in the insulin signaling cascade has been associated with severe forms of insulin resistance and type 2 diabetes [7]. Currently, metformin is considered the first line therapeutic treatment for hyperglycemia and diabetes [8]. 

Approximately 30% of patients taking metformin develop nausea and bloating with abdominal pain, and 5–10% of patients are unable to continue on the medication [9]. Thus, screening and finding natural active compounds that could ameliorate insulin resistance and glucose metabolism is necessary. 

Carpachromene is a natural bioactive compound that was isolated from the ethyl acetate fraction of fresh leaves of *Ficus benghalensis* [10]. Carpachromene was previously reported to exhibit cytotoxicity against HepG2, PLC/PRF/5 and Raji cancer cell lines [11]. It also induced apoptosis in human ovarian cancer cells, SW 626, by decreasing mitochondrial smac and increasing cytosolic smac [12]. The antimycobacterial activity of carpachromene was investigated, but it showed no activity against *Mycobacterium tuberculosis* H_37_R_V_ in vitro [13]. In addition, carpachromene was examined as an antibrowning agent, but it showed no tyrosinase inhibition activity [14].

Looking for new pharmacological activities for existing candidates became essential [15,16,17,18,19,20,21,22]. Herein, we investigated, for the first time, the potential activity of carpachromene, which was shown to inhibit α-glucosidase enzyme activity [23], on glucose consumption, metabolism, and insulin signaling in a HepG2 cells insulin resistant model.

## 2. Results

### 2.1. Insulin Resistant Model (IRM) Development

**Figure 1:**
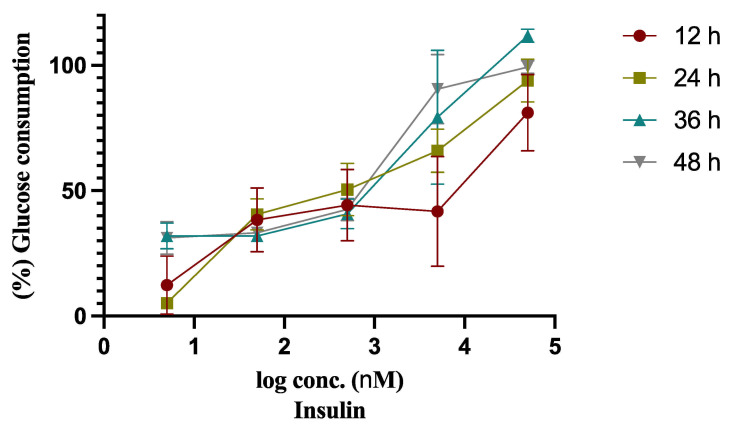
Establishment of insulin resistant model on HepG2 cells through examining the effect of different concentrations of insulin (0.005, 0.05, 0.5, 5, 50 µM) on glucose consumption after 12, 24, 36, 48 h, when compared to control non-insulin treated HepG2 cells. Bars represent mean ± SD.

To obtain the insulin resistant model of HepG2 cells (HepG2/IRM), we examined the effect of different concentrations of insulin (0.005, 0.05, 0.5, 5, 50 µmol) on glucose concentration for different time intervals 12, 24, 36, 48, as shown in Table 1 and Figure 1. As shown in Figure 1, cells incubated with 0.005 µM of insulin for 24 h showed the lowest cellular glucose consumption when compared to the control cells without insulin treatment. Therefore, treatment of HepG2 cells with 0.005 µM of insulin for 24 h was finalized to conduct HepG2/IRM cells. 

### 2.2. Cell Viability Assay

**Figure 2:**
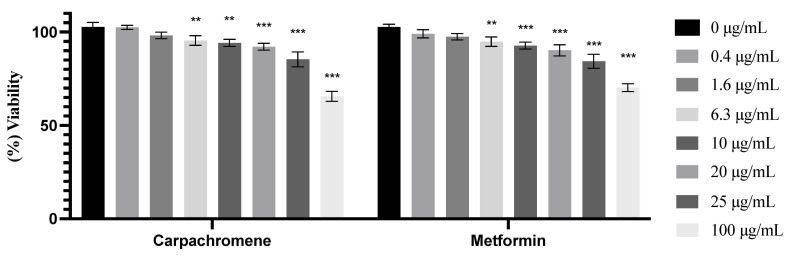
Cell viability after treatment with different concentrations (0.4, 1.6, 6.3, 10, 20, 25, 100 µg/mL) of carpachromene or metformin on HepG2/IRM cells for 48 h. Bars represent mean ± SD. Significant difference was analyzed by two-way ANOVA test followed by post hoc Dunnett test, where ** *p* < 0.01, *** *p* < 0.001, compared to untreated HepG2/IRM cells.

HepG2/IRM cells were treated with different concentrations of carpachromene or metformin (0.4, 1.6, 6.3,10, 20, 25, 100 µg/mL) for 48 h to examine their cytotoxic activity on the cells. As shown in Figure 2, a significant (*p* < 0.01) decrease in cell viability started to occur from 6.3 µg/mL concentration of carpachromene or metformin on HepG2/IRM cells. However, according to Dewi et al., further examination can be preceded with compounds that show over 90% cell viability [24]. At the concentrations of 6.3, 10, 20 µg/mL carpachromene decreased (*p* < 0.01) cell viability to 95.46% ±2.57, 94.18% ±1.91, and 92.19% ±1.82, respectively. At the concentrations of 25 and 100 µg/mL, carpachromene significantly (*p* < 0.01 and *p* < 0.001, respectively) decreased cell viability to 85.43% ± 4.01 and 65.58% ± 2.67, respectively, when compared to untreated HepG2/IRM cells.

### 2.3. Effect of Carpachromene on Glucose Concentration

**Figure 3:**
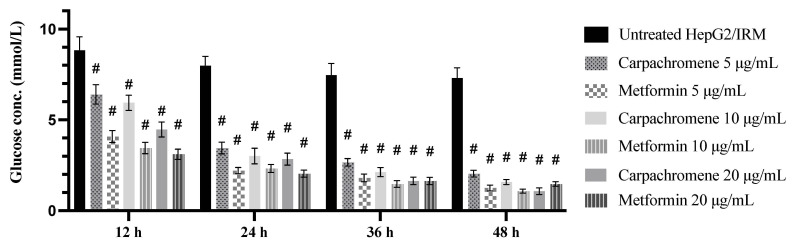
Glucose concentration in the media after treatment of HepG2/IRM cells with carpachromene or metformin at the concentrations of 5, 10, and 20 µg/mL for 12, 24, 36, and 48 h. Bars represent mean ± SD. Significant difference was analyzed by two-way ANOVA test followed by post hoc Dunnett test, where # *p* < 0.001, compared to untreated HepG2/IRM cells.

HepG2/IRM cells were treated with different concentrations of carpachromene (5, 10, and 20 µg/mL) for different time intervals (12, 24, 36 and 48 h). Metformin was used as a positive control. Our findings revealed that carpachromene decreased glucose concentrations in the media in a concentration- and time-dependent manner (Figure 3). At the concentration of 5 µg/mL, carpachromene significantly (*p* < 0.001) decreased glucose concentration to 6.4 ± 0.53 mmol, 3.45 ± 0.32 mmol/L, 2.66 ± 0.21 mmol/L, and 2.04 ± 0.18 mmol/L after 12, 24, 36, and 48 h treatment, respectively, when compared to untreated HepG2/IRM cells. In addition, at the concentration of 10 µg/mL, carpachromene significantly (*p* < 0.001) decreased glucose concentration to 5.94 ± 0.42 mmol/L, 3.01 ± 0.43 mmol/L, 2.12 ± 0.25 mmol/L, and 1.58 ± 0.14 mmol/L after 12, 24, 36, 48 h treatment, respectively, when compared to untreated HepG2/IRM cells. Furthermore, at the concentration of 20 µg/mL, carpachromene significantly (*p* < 0.001) decreased glucose concentration to 4.47 ± 0.41 mmol/L, 2.84 ± 0.33 mmol/L, 1.64 ± 0.21 mmol/L, and 1.07 ± 0.18 mmol/L after 12, 24, 36, and 48 h treatment, respectively, when compared to untreated HepG2/IRM cells. 

### 2.4. Carpachromene Improved Cellular Glycogen Synthesis in HepG2/IRM Cells

**Figure 4:**
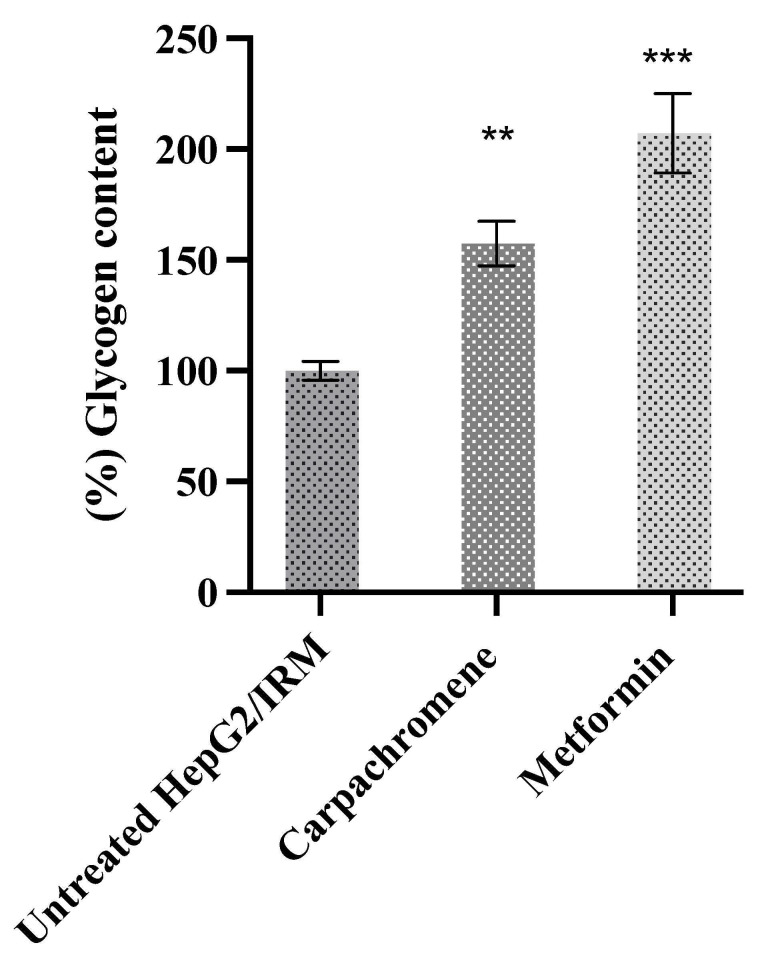
Glycogen content before and after treatment of HepG2/IRM cells with carpachromene or metformin (20 µg/mL), where untreated HepG2/IRM cells were considered 100%. Bars represent mean ± SD. Significant difference was analyzed by one-way ANOVA test followed by post hoc Dunnett test, where ** *p* < 0.01, *** *p* < 0.001, compared to control untreated HepG2/IRM cells.

To investigate the effect of carpachromene on the glycogenesis pathway, we examined the intracellular glycogen content in control untreated HepG2/IRM cells and carpachromene-treated (20 µg/mL) HepG2/IRM cells. Metformin was used as a positive control. As shown in Figure 4, the intracellular glycogen content was significantly (*p* < 0.01) increased to 157.43% ± 10.03, when compared to untreated HepG2/IRM cells. 

### 2.5. Carpachromene Modulated the Expression of Proteins Involved in Insulin Signaling

**Figure 5:**
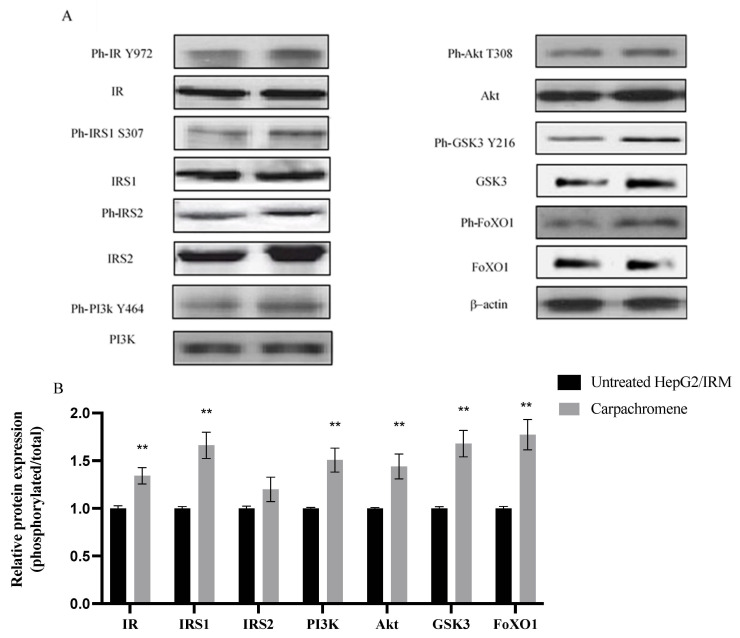
Effect of Carpachromene on the expression of phosphorylated and total proteins of IR, IRS1, IRS2, PI3K, Akt, GSK3, and FoxO1 proteins in HepG2/IRM cells. (**A**) Representative Western blots of phosphorylated and total proteins of IR, IRS1, IRS2, PI3K, Akt, GSK3, and FoxO1 in HepG2/IRM cells before (left lane) and after (right lane) treatment with 20 µg/mL carpachromene. β-actin was used as internal loading control. (**B**) Phosphorylated/total protein expression ratio in HepG2/IRM cells relative to untreated HepG2/IRM cells, after normalization to the corresponding β-actin protein expression. Bars represent mean ± SD. Significant difference was analyzed by student t test, where ** *p* < 0.01, compared to untreated HepG2/IRM cells.

To understand the mechanism of action of carpachromene, we examined the phosphorylated and total protein expression of IR, IRS1, IRS2, PI3K, Akt, Glycogen synthase kinase (GSK3), and forehead box protein O1 (FoxO1) proteins. β-actin was used as an internal control. As shown in Figure 5, treatment of HepG2/IRM cells with 20 µg/mL carpachromene significantly (*p* < 0.01) increased the protein expression ratios of IR, IRS1, PI3K, Akt, GSK3, and FoxO1 when compared to untreated HepG2/IRM cells. On the other hand, there was no significant difference (*p* > 0.05) in IRS2 expression ratio before and after the treatment with carpachromene.

### 2.6. Carpachromene Modulated Hepatic Enzymes Activity

**Figure 6:**
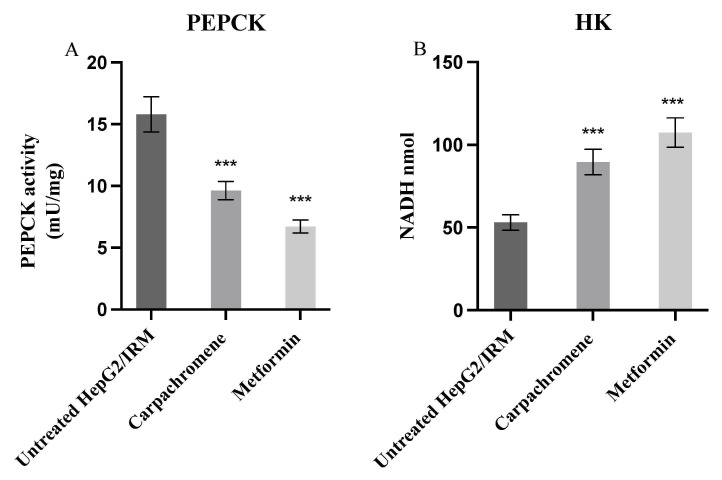
Effect of carpachromene (20 µg/mL) on the activity of (**A**) PEPCK and (**B**) HK enzymes on HepG2/IRM cells. Metformin was used as positive control. Bars represent mean ± SD (*n*= 3), significant difference was analyzed by one-way ANOVA followed by Dunnett test, *** *p* < 0.001, when compared to untreated HepG/IRM cells.

To examine the effect of carpachromene on the activity of hepatic metabolic enzymes, phosphoenolpyruvate carboxykinase (PEPCK) and Hexokinase (HK) hepatic enzyme activities were investigated. Metformin was used as a positive control. After treatment of 20 µg/mL carpachromene on HepG2/IRM cells, PEPCK enzyme activity was significantly (*p* < 0.001) decreased to 9.63 mU/mg ± 0.743 and the HK enzyme activity (represented as the amount of nicotinamide adenine dinucleotide (NADH) obtained from the reaction) was significantly (*p* < 0.001) increased to 89.6 nmol ±7.66, when compared to untreated HepG2/IRM cells, as shown in Figure 6.

## 3. Discussion

Liver is a major insulin target organ which plays a predominant role in glucose and lipid metabolism. The direct insulin action on hepatocytes controls several metabolic pathways including glycolysis, glycogenesis, and gluconeogenesis [25]. The impairment of insulin action on its receptor results in a condition of insulin resistance which is associated by an increase in beta cell insulin production and hyperinsulinemia [26]. Hepatoma cell lines, particularly HepG2 cells, have been previously used to model hepatocytes in glucose metabolism and insulin signaling. Several studies induced an insulin resistant state in HepG2 cells to examine the antidiabetic activity of different compounds on hepatocytes [8,27,28]. Sefried et al. indicated that HepG2 cells were shown to have a gluconeogenic and hepatic-gene expression pattern similar to that of the in vivo conditions [29]. The present study established an insulin resistant model on HepG2 cells as previously reported [30]. Different concentrations of insulin were examined at different time intervals to obtain the optimum time and concentration that showed the lowest glucose consumption. Our findings revealed that 0.005 µM of insulin treatment for 24 h exerted the lowest glucose consumption, so we utilized it to obtain the insulin-resistant state of HepG2 cells for further analysis of carpachromene activity on insulin signalling and glucose metabolism. 

Natural agents exhibited many in vitro [31,32] and in vivo [33,34] pharmacological actions in various disorders like carcinogenesis, [35] fibrosis, [36,37] angiogenesis, [38] and immunomodulation [39]. Several natural products have been examined for their hypoglycaemic activity against the insulin resistant model of HepG2 cells. According to Bian et al., natural triterpenoids isolated from *Akebia trifoliata* stem exerted inhibitory activity against α-glucosidase enzyme. These compounds showed no cytotoxicity from 6.25–25 µM against HepG2/IRM, while toxic activity started to occur at higher doses [40]. A study by Yang et al. indicated that baicalein, a natural bioactive compound, showed cytotoxicity on HepG2 cells at concentration of 10^−4^ mol/L [41]. Another study by Li et al. examined the activity of glycosides against HepG2 resistant cells where these compounds showed cytotoxity at 200 and 250 µM [42]. 

Carpachromene is a natural flavonoid that can be found in various medicinal plants including *Ficus nervosa*, *Calophyllum symingtonianum*, *Flindersia pimenteliana*, *Ficus formosana*, and *Ficus benglanesis* [10,11,13,23,43]. Carpachromene was observed to have anti-plasmodial activity, a study by Robertson et al. indicated that carpachromene has shown anti-plasmodial principles against chloroquine-sensitive (3D7) and chloroquine-resistant (Dd2) *Plasmodium falciparum* with no cytotoxicity against HEK-293 cells at 40 µM [11]. It exerted cytotoxic activity against HepG2, PLC/PRF/5 and Raji cancer cell lines that was indicated by XTT assay [11]. Furthermore, it modulated the expression of Smac protein resulting in induction of apoptosis for ovarian cancer cells [12].

To ensure the safety of carpachromene treatment on HepG2/IRM cells, cell viability assay was conducted. At the concentrations of 6.3, 10, 20 µg/mL of carpachromene treatment, cell viability assay showed more than 90% of viable HepG2/IRM cells. Hence, carpachromene was used at 5, 10, 20 µg/mL for further analysis. Then, glucose concentration was estimated after carpachromene treatment at different time intervals. Our findings revealed a substantial decrease in glucose levels in the media indicating the increase of glucose uptake and consumption in a time- and concentration-dependant manner. In addition, the increase in glycogen synthesis was notably shown after carpachromene treatment in comparison to untreated HepG2/IRM cells. These results prompted the investigation of carpachromene activity on HepG2/IRM cells at the molecular level. 

PI3K/Akt pathway plays a vital role in the metabolism inside the body. The Akt family was shown to regulate glycolysis, glycogenesis, and gluconeogenesis pathways in hepatocytes. Akt2, which is mainly expressed in insulin-responsive tissues, stimulates the expression of glucose transporter 4 (GLUT4) to increase glucose uptake by the cells. Akt promotes glycolysis and energy production via stimulation of HK enzyme to convert glucose to glucose-6-phosphate. In addition, Akt promotes glycogen production through the phosphorylation and inhibition of GSK3 which primarily inhibits glycogen synthase enzyme, a key enzyme in glycogen synthesis [44]. Another important protein in insulin signalling is FoxO1 protein, which is one of the main targets of Akt that was found to increase the expression of PEPCK and glucose-6-phosphatase enzymes. Akt primarily phosphorylates and inhibits FoxO1 proteins with further inhibition on gluconeogenesis pathway [45]. In the present study, carpachromene treatment of insulin resistant HepG2 cells increased the expression ratio of IR, IRS1, PI3K, Akt, GSK3, and FoxO1 proteins. We speculate that carpachromene increased the expression ratio of insulin receptor and IRS1 with further down-stream activation of PI3K/Akt pathway to stimulate glucose uptake and glycolysis.

Alternatively, the activation of the PI3K/Akt pathway led to the inhibition of the GSK3 enzyme with further increasing glycogen synthase activity and glycogenesis. In addition, the PI3K/Akt pathway inhibited the FoxO1 protein leading to the inhibition of PEPCK and glucose-6-phosphatase enzymes resulting in the inhibition of the gluconeogenesis pathway. Finally, carpachromene showed no significant activity on the expression ratio of IRS2. 

In summary, carpachromene is a natural active compound that was previously shown to inhibit α-glucosidase enzyme activity. It increased glucose consumption and uptake in a concentration- and time- dependant manner, and it also increased glycogen content in HepG2/IRM. On a molecular basis, carpachromene stimulated the phosphorylation of the insulin receptor and IRS1 proteins, which consequently activated the PI3K/Akt pathway. PI3K/Akt centrally regulates the metabolic pathway through the increase of glucose uptake via glucose transporter, GLUT4, and the increase of hexokinase enzyme activity to stimulate glycolysis. Furthermore, PI3K/Akt stimulates glycogenesis through the phosphorylation and inhibition of GSK3 enzyme. Additionally, PI3K//Akt inhibits gluconeogenesis through the phosphorylation and inhibition of FoxO1 protein. The present study proposed, for the first time, the antidiabetic activity of carpachromene via the IR/IRS1/PI3K/Akt/GSK3/FoxO1 pathway. 

## 4. Materials and Methods

### 4.1. Isolation of Carpachromene

**Figure 7:**
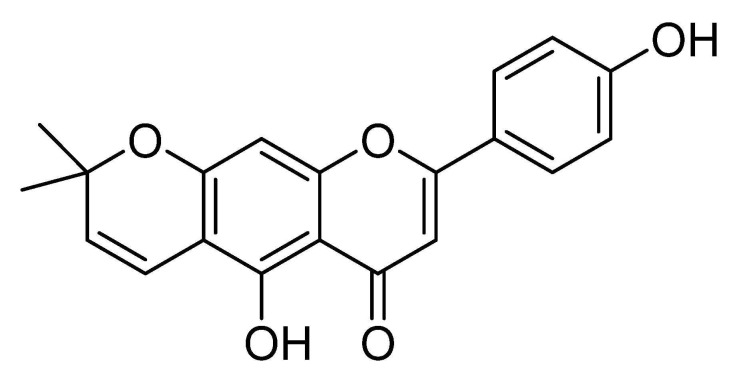
Chemical structure of carpachromene extracted from the ethyl acetate fraction of fresh leaves of *Ficus benghalensis*.

Carpachromene was isolated as previously described [10]. Briefly, it was extracted from the ethyl acetate fraction of fresh leaves of *Ficus benghalensis* and identified by ^1^H-NMR and ^13^C-NMR spectral data, shown in the Supplementary Data. The chemical structure of carpachromene is shown in Figure 7.

### 4.2. Cell Culture and Induction of Insulin Resistant HepG2 Cell Model

HepG2 cells were obtained from American Type Culture Collection (ATCC, Manassas, VA, USA). Fresh Dulbecco’s Modified Eagle’s Medium (DMEM, Sigma-Aldrich, Inc, St. Louis, MO, USA) was used as a culture medium, augmented with 10% foetal bovine serum (FBS, Biosolutions International, Melbourne, Australia), 100 U/mL Penicillin and 1% streptomycin (Invitrogen, Grand Island, NY, USA) in a humidified 5% CO_2_ atmosphere at 37 °C. 

Insulin resistant HepG2 cell model was accomplished as described [30]. In brief, cells were cultured in FBS-free medium for 6 h, then treated with insulin (Sigma-Aldrich Inc., St. Louis, MO, USA) at concentrations of 0.005, 0.05, 0.5, 5, and 50 µM for different time intervals of 12, 24, 36, and 48 h in three independent repeats. Glucose concentration was assayed in 10 µL of the media utilizing glucose assay kit (Signa-Aldrich Inc.) according to the manufacturer’s instructions. The percentage of cellular glucose consumption was determined as the difference between glucose concentration before and after insulin treatment relative to control non-insulin treated cells.

The optimum incubation time and insulin concentration for conducting the insulin resistant model (IRM) were determined. The finalized insulin resistant HepG2 cells were called HepG2/IRM.

### 4.3. Cell Viability Assay

Cell viability assay was achieved using MTT reagent [3-(4, 5-dimethyl thiazol-2yl)-2, 5-diphenyltetrazolium bromide] (Sigma Aldrich, Inc, St. Louis, MO, USA). After HepG2/IRM cells were obtained, 10,000 cells were seeded per well in triplicates in 96-well plates. Then, the cells were treated with different concentrations (0.4, 1.6, 6.3, 10, 20, 25, and 100 µg/mL) of carpachromene or metformin. After incubation for 48 h, 10 µL of MTT (5 µg/mL) was added per well and incubated in the dark for 3 h at 37 °C. To dissolve the Formazan crystals that were formed, 100 µL of DMSO were used and absorbance was measured using an ELISA reader (Multiskan ^TM^ FC microplate photometer, ThermoFisher Scientific, Life technologies Ltd., Paisley, UK) at 570 nM as described [46].

### 4.4. Carpachromene Effect on Glucose Concentration in HepG2/IRM

HepG2/IRM cells were obtained, and 10,000 cells were seeded in 96 well-plates in triplicates. Then, cells were treated with non-toxic concentrations of carpachromene (5, 10, 20) µg/mL for different durations. Metformin was used as a positive control. Glucose concentration was assayed in 10 µL of the medium after 12, 24, 36, and 48 h of treatment using glucose assay kit (Sigma-Aldrich, Inc., St. Louis, MO, USA) according to the manufacturer’s instructions [27].

### 4.5. Determination of Intracellular Glycogen Content

To examine the effect of carpachromene on intracellular glycogen, HepG2/IRM cells were seeded into a 6-well plate at a density of 3 × 10^5^ cells/well. Cells were treated with 20 µg/mL carpachromene for 24 h. Metformin was used as a positive control. Then, cells were washed three times with PBS. Glycogen assay kit (#ab65620, Abcam, Cambridge, UK) was utilized according to the manufacturer’s instructions. Microplate reader (ROBONIK P2000 ELISA Reader, OBONIK, Thane, India) was used to measure absorbance at 570 nm. Additionally, the protein content of HepG2/IRM cells was quantified by Bradford method [47], and the values were showen as a ratio of glycogen (mg)/protein (mg). Then, a percentage ratio was calculated where untreated HepG2/IRM cells were considered 100%.

### 4.6. Analysis of Protein Expression

The present study investigated the protein expression levels of phosphorylated insulin receptor (ph-IR) (#ab5678, Abcam), total insulin receptor (IR) (#ab137747, Abcam), phosphorylated IRS1 (ph-IRS1; #ab1194), total IRS1 (IRS1; ab40777), phosphorylated IRS2 (ph-IRS2; #07-1517, Sigma Aldrich Inc.), total IRS2 (RS2) (#ab245386, Abcam), phosphorylated PI3K (ph-PI3K) (#ab138364, Abcam), total PI3K (#ab154598), phosphorylated Akt (ph-Akt; #ab38449, Abcam), total Akt (Akt) (#ab188099, Abcam), phosphorylated GSK3 (ph-GSK3; #ab75745, Abcam), total GSK3 (#ab131356, Abcam), phosphorylated FoxO1 (ph-FoxO1; #ab131339, Abcam), and total FoxO1 (FoxO1; #ab39670, Abcam). Sodium dodecyl sulphate–polyacrylamide gel electrophoresis (SDS-PAGE) analysis was performed. HepG2/IRM cells were treated with 20 µg/mL of carpachromene for 24 h. After washing with PBS, the protein extraction was performed in RIPA lysis buffer, containing 50 mM Tris–Cl, pH 7.5; 0.1% SDS, 150 mM NaCl, 0.5% Sodium deoxycholate, 1 mM PMSF, and 1% Nonidet P-40, supplemented with the complete protease inhibitor cocktail (Roche, Mannheim, Germany). The Bradford method was used to determine the protein concentration [47]. Cell lysates containing 30 µg protein were separated by SDS-PAGE (15% acrylamide), transferred to a Hybond™ nylon membrane (GE Healthcare) and incubated for 1 h at room temperature in Blocking Solution. Membranes were incubated overnight at 4 °C with ph-insulin receptor, ph-insulin substrate1, ph-insulin substrate2, ph-Akt, and ph-PI3K antibodies diluted (1:1500) with PBS. Then, membranes were washed for 30–60 min and incubated at room temperature for 1 h with the HRP-conjugated secondary antibody (New England Biolabs) diluted (1:1500) in PBS [48]. According to the manufacturer’s instructions, immunoreactive proteins were detected using an enhanced chemiluminescence kit (GE Healthcare, Little Chalfont, UK) by a luminescent image analyzer (LAS-4000, Fujifilm Co., Tokyo, Japan). An antibody against β-actin (New England Biolabs, Hertfordshire, England) (1:1000) was used to detect β-actin, which was used as a loading control. Electrophoresis and electroblotting, using a discontinuous buffer system, were carried out in a Bio-Rad Trans-Blot SD Cell apparatus (Bio-Rad, Hercules, CA, USA). Densitometric analysis was then performed by using The Image Processing and Analysis Java (ImageJ, 1.8.0_172) program. Data were normalized to β-actin levels.

### 4.7. Effect of Carpachromene on Hepatic Enzymes

To examine the effect of carpachromene on PEPCK and HK hepatic enzymes activity, HepG2/IRM cells were seeded into a 6-well plate at a density of 3 × 10^5^ cells per well. Cells were treated with 20 µg/mL carpachromene for 24 h. Metformin was used as a positive control. Then, cells were washed three times with PBS. PEPCK assay kit (#ab239714, Abcam, Waltham, MA, USA) and Hexokinase activity assay kit (#ab136957, Abcam, Waltham, MA, USA) were utilized according to the manufacturer’s instructions. A standard curve was obtained, and the absorbance was measured at 570 nm and 450 nm for PEPCK and HK, respectively, using an ELISA plate reader. The activities of PEPCK and HK were calculated from the standard curves. The experiments were performed in triplicates. 

### 4.8. Statistical Analysis

At least three independent experiments were used to obtain the results. Data were expressed as mean ± standard deviation. One or two-way analysis of variance (ANOVA) followed by post hoc Dunnett test were used to analyse the differences of multiple comparison using GraphPad Prism 9 statistical software (GraphPad) and Excel software (Microsoft, Redwood, WA, USA). Differences were considered significant when the probability values (P) were less than 0.05.

## 5. Conclusions

This study examined, for the first time, the potential antidiabetic activity of carpachromene on a biochemical and molecular level. Our findings revealed that carpachromene showed central molecular regulation of glucose metabolism and insulin signalling via IR/IRS1/ PI3K/Akt/GSK3/FoxO1 pathway.

## Figures and Tables

**Table 1:** Effect of different concentrations of insulin on glucose consumption in HepG2 cells at different time intervals to conduct IRM.

Insulin Conc. (µM)	Glucose Consumption (mmol/L)			
	12 h	24 h	36 h	48 h
-	3.577 ± 0.199	4.441 ± 0.087	5.477 ± 0.045	6.200 ± 0.092
0.005	0.432 ± 0.406 ***	0.229 ± 0.077 ***	1.750 ± 0.270 ***	1.932 ± 0.412 ***
0.05	1.378 ± 0.477 ***	1.803 ± 0.310 ***	1.750 ± 0.026 ***	2.062 ± 0.139 ***
0.5	1.563 ± 0.445 ***	2.248 ± 0.502 ***	2.232 ± 0.310 ***	2.636 ± 0.284 ***
5	1.507 ± 0.841 ***	2.933 ± 0.434 **	4.345 ± 1.462 *	5.620 ± 0.923
50	2.916 ± 0.670	4.175 ± 0.430	6.124 ± 0.114	6.158 ± 0.094

Values represent mean ± SD from three independent repeats. Significant difference was analyzed by two-way ANOVA test followed by post hoc Dunnett test, where * *p* < 0.5, ** *p* < 0.01, *** *p* < 0.001, compared to control non-insulin-treated cells.

## Data Availability

All data are fully available and included in the manuscript.

## Supplementary Materials

The following are available online, Figure S1: ^1^H NMR spectrum of Carpachromene, Figure S2: ^13^C NMR spectrum of Carpachromene, Table S1: ^1^H-NMR spectral data of Carpachromene (CD_3_OD, 600 MHz), Table S2: ^13^C-NMR spectral data of Carpachromene (CD_3_OD, 150 MHz).

## Sample Availability Statement

Samples of the compound are not available from the authors.
