# Supplementary material for: Carpachromene Ameliorates Insulin Resistance in HepG2 Cells via Modulating IR/IRS1/PI3k/Akt/GSK3/FoxO1 Pathway
Source: Molecules. 2021 Dec 16;26(24):7629. doi: 10.3390/molecules26247629 (PMC8708443; doi:10.3390/molecules26247629)
Supplement: Supplementary file 1 [file molecules-26-07629-s001.zip › molecules-1485513-supplementary.pdf]

NMR data of Carpachromene

Table S1. ^{1}H-NMR spectral data of Carpachromene (CD_3OD, 600 MHz).

Chemical shift (δ) ppm	Multiplicity	*J* constant	Assignment
		8.76	
7.86 (2H)		8.76	H – 2` and H-6`H – 3` and H-5`
6.93 (2H)		10.02	H – 4``
6.68 (1H)	*ddddsss*	10.02	H - 3``H - 8
5.72 (1H)6.47 6.61		------	H - 3
1.46 (6H)		------	Two methyl groups

Table S2. ^{13}C-NMR spectral data of Carpachromene (CD_3OD, 150 MHz).

CarbonNo.	Chemical shift (δ) ppm	CarbonNo.	Chemical shift (δ) ppm
2	160.99	1`	123.18
3	106.11	2`,6`	129.58
4	184.11	3`,5`	117.12
5	158.59	4`	166.48
6	106.72	2``	79.28
7	162.98	3``	129.69
8	96.15	4``	116.14
9	157.26	2CH3	28.59
10	103.97		

Mass spectral analysis:

The ESI (-ve mode) of Carpachromene showing peaks at m/z: 335.1 [M^-] whichis equivalent to $C_{20}H_{16}O_5$

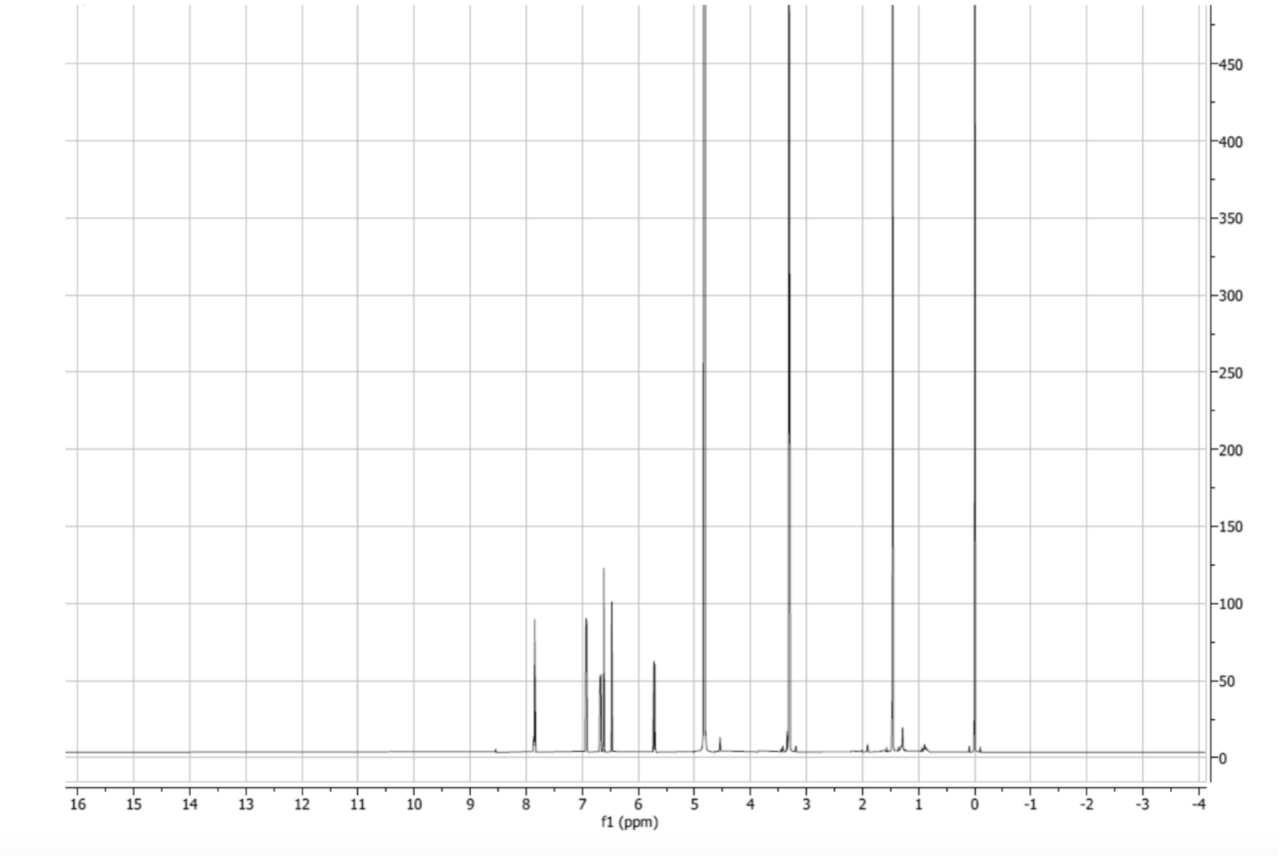

Figure S1. ^{1}H NMR spectrum of Carpachromene.

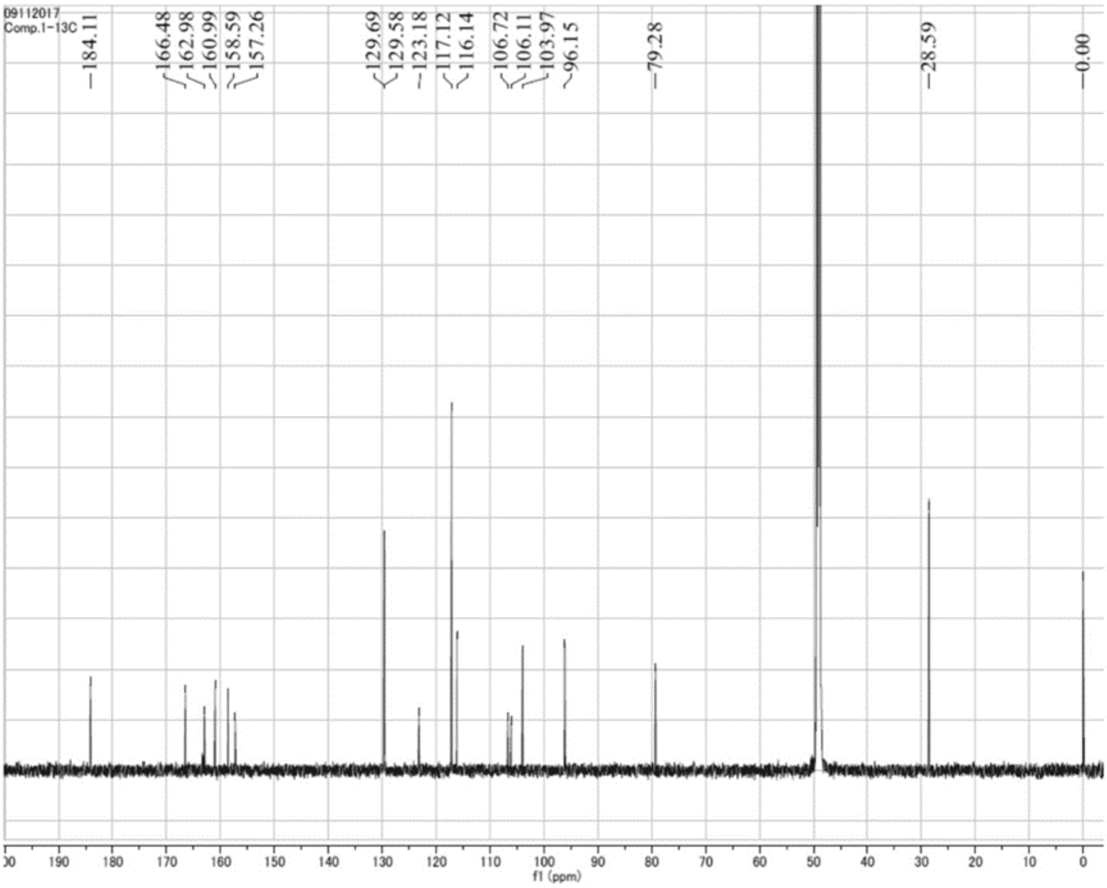

Figure S2. ^{13}C NMR spectrum of Carpachromene.
